# Retraction: Lithological information extraction and classification in hyperspectral remote sensing data using Backpropagation Neural Network

**DOI:** 10.1371/journal.pone.0301149

**Published:** 2024-03-20

**Authors:** The PLOS ONE Editors

The *PLOS ONE* Editors retract this article [1] because it was identified as one of a series of submissions for which we have concerns about peer review integrity and similarities across articles. These concerns call into question the validity and provenance of the reported results. We regret that the issues were not identified prior to the article’s publication.

The authors either did not respond directly or could not be reached.
